# Increased Oxidative Metabolism and Neurotransmitter Cycling in the Brain of Mice Lacking the Thyroid Hormone Transporter Slc16a2 (Mct8)

**DOI:** 10.1371/journal.pone.0074621

**Published:** 2013-10-01

**Authors:** Tiago B. Rodrigues, Ainhoa Ceballos, Carmen Grijota-Martínez, Barbara Nuñez, Samuel Refetoff, Sebastian Cerdán, Beatriz Morte, Juan Bernal

**Affiliations:** 1 Instituto de Investigaciones Biomedicas, Consejo Superior de Investigaciones Cientificas and Universidad Autonoma de Madrid, Madrid, Spain; 2 CRUK, Cambridge Institute and Department of Biochemistry, University of Cambridge, Cambridge, United Kingdom; 3 Center for Biomedical Research on Rare Diseases, Madrid, Spain; 4 Departments of Medicine, Pediatrics and Genetics, University of Chicago, Chicago, Illinois, United States of America; University Claude Bernard Lyon 1, France

## Abstract

Mutations of the monocarboxylate transporter 8 (MCT8) cause a severe X-linked intellectual deficit and neurological impairment. MCT8 is a specific thyroid hormone (T_4_ and T_3_) transporter and the patients also present unusual abnormalities in the serum profile of thyroid hormone concentrations due to altered secretion and metabolism of T_4_ and T_3_. Given the role of thyroid hormones in brain development, it is thought that the neurological impairment is due to restricted transport of thyroid hormones to the target neurons. In this work we have investigated cerebral metabolism in mice with Mct8 deficiency. Adult male mice were infused for 30 minutes with (1-^13^C) glucose and brain extracts prepared and analyzed by ^13^C nuclear magnetic resonance spectroscopy. Genetic inactivation of *Mct8* resulted in increased oxidative metabolism as reflected by increased glutamate C4 enrichment, and of glutamatergic and GABAergic neurotransmissions as observed by the increases in glutamine C4 and GABA C2 enrichments, respectively. These changes were distinct to those produced by hypothyroidism or hyperthyroidism. Similar increments in glutamate C4 enrichment and GABAergic neurotransmission were observed in the combined inactivation of *Mct8* and *D2*, indicating that the increased neurotransmission and metabolic activity were not due to increased production of cerebral T_3_ by the *D2*-encoded type 2 deiodinase. In conclusion, Mct8 deficiency has important metabolic consequences in the brain that could not be correlated with deficiency or excess of thyroid hormone supply to the brain during adulthood.

## Introduction

Thyroid hormones [3,5,3',5'-tetraiodothyronine (T_4_) and 3,3',5-triiodothyronine (T_3_)] play an important role in brain development and function [1], [2]. Thyroid hormones uptake by the target cells is facilitated by several forms of plasma membrane transporters [3]. The monocarboxylate transporter 8 (MCT8, SLC16A2) is very specific for the transport of T_4_ and T_3_ [4], and plays an essential role in their uptake by the brain. MCT8 is expressed in the blood-brain barrier (BBB), the choroid plexuses and the plasma membrane of neural cells [5]. *MCT8* gene mutations cause an X-linked syndrome combining severe neurodevelopmental impairment and abnormal distribution and metabolism of thyroid hormones [6]–[18]. The syndrome manifests in infants as early as two months of age. It is characterized by truncal hypotonia evolving into spastic quadriplegia, mental retardation with severe global developmental delay, dystonic movements, lack of speech development and other signs of neurological impairment. The defective transport of thyroid hormones across cell membranes also causes decreased serum concentrations of T_4_ and 3,3',5'-triiodothyronine (reverse T_3_, rT_3_), and increased T_3_ by a complex mechanism involving hormone secretion, metabolism and excretion [11], [14], [19]–[21].

It is assumed that the neurological impairment is due to severe thyroid hormone deprivation to neurons during a critical phase of development. In agreement with brain hypothyroidism is the finding of delayed myelination on magnetic resonance imaging [13], [16]. *In vivo* ^1^H nuclear magnetic resonance (NMR) spectroscopy has also shown altered choline and *N*-acetylaspartate concentrations similar to those found in congenital hypothyroidism [18]. However, the clinical picture of *MCT8* gene mutations does not resemble other conditions due to profound thyroid hormone deficiency during development. For example, neurological cretinism is characterized by mental retardation, deafness and deaf mutism, pyramidal and extrapyramidal dysfunction, and a specific gait disorder [22], [23]. This contrasts with the essential features of patients with MCT8 mutations, which according to Schwartz and Stevenson [17] are “congenital hypotonia, severe cognitive impairment, muscle weakness and abnormal hand positioning.” The underlying mechanism of neuronal damage in MCT8 mutations remains unknown. In particular, in the cellular contexts, MCT8 may perform some functions besides thyroid hormone transport [24].

In contrast to the severe neurological impairment of patients with MCT8 mutations, only some changes of performance in some behavioral tests have been reported in *Mct8KO* mice [25]. *Mct8* inactivation in mice reproduces the changes of circulating thyroid hormone concentrations observed in the patients, and brain T_3_ content is reduced [26], [27]. However the mice do not present structural changes in brain that might indicate hypothyroidism. In addition, the expression of most thyroid hormone-dependent genes in the cerebral cortex is normal with only a few exceptions [28]. Differences between the human and mouse phenotypes might be due to differences in expression of the specific T_4_ transporter OATP1C1/Oatp1c1 [29], which is expressed in the mouse brain capillaries and plasma membrane of the astrocytic end-feet [5], but has low expression in primates [30]. Oatp1c1 would allow T_4_ transport through the mouse BBB, even in the absence of Mct8, leading to the production of local brain T_3_ by the type 2 deiodinase (D2), thus preventing Central Nervous System damage.

Still, the impact of *Mct8* inactivation on brain metabolism and neurotransmitter balance has not been analyzed. In the present work we performed metabolic studies in the brain of *Mct8KO* mice using ^13^C NMR spectroscopy. This approach provides comprehensive information on cerebral energetics and metabolism [31]. In particular, ^13^C NMR spectroscopy determination of the ^13^C label incorporated in relevant carbons of glutamate, glutamine and GABA after (1-^13^C) glucose infusions was shown to reveal the activities of the cerebral tricarboxylic acid (TCA) cycles and the transcellular glutamate-glutamine and glutamate-GABA cycles [32]. We have used *Mct8KO* mice with deficient T_3_ transport to the brain [26], and mice with disruption of the *D2* gene (*D2KO*) and therefore unable to generate T_3_ from T_4_ locally in the brain [33]. Data were compared with those from mice made hypothyroid or hyperthyroid by pharmacological means. We found that the *Mct8KO* mice present paradoxical metabolic alterations that cannot be explained solely by deficiency or excess of thyroid hormone supply to the brain.

## Methods

### Ethics Statement

All experimental procedures involving animals were performed following the European Union Council guidelines (directive 2010/63/UE) and Spanish regulations (R.D.1201/2005, and Law 32/2007), and were approved by the Subcommittee on Bioethics of the Consejo Superior de Investigaciones Científicas (CSIC) with the approval reference numbers SAF2008-01168 and SAF2011-25608. All efforts were made to minimize suffering. Anesthetics were used as indicated.

### Chemicals

(1-^13^C) glucose (99% ^13^C) was obtained from Cambridge Isotope Laboratories (Andover, MA, USA). ^2^H_2_O (99.9% ^2^H) was acquired from Apollo Scientific Ltd. (Stockport, Cheshire, UK). All the other items were of the highest purity available commercially from Sigma-Aldrich (Madrid, Spain).

### Animals and genotypes

All experiments were performed with animals 6–7 months of age. Animals were housed under temperature (22±2°C) and light (12:12 light-dark cycle; lights on at 7 a.m.) controlled conditions and had free access to food and water. *Mct8KO* (genotype *Mct8^−/y^*) mice were generated by Dumitrescu *et al* [26] using homologous recombination. Experiments were carried out on *WT* and *KO* male litter mates derived from heterozygous females crossed with WT males of the C57BL/6J strain. Genotypes were confirmed by PCR of tail DNA (38 cycles at 61°C annealing temperature) using the following primers: Forward common: 5'-ACAGAGCAAGTTCCAAGACA-3'; reverse *WT*-specific: 3'-ATAGAAATCAGGCTTGGGAG-5'; reverse *KO*-specific: 3'-TTTGTCCTTACGCTGCTCTC-5'. Using this procedure, the *WT* allele generated a 573 base pairs (bp) PCR product and the null allele a 325 bp product. Male mice deficient in D2 (*D2KO*; genotype *D2^−/−^*) [33] were crossed with Mct8-deficient heterozygous females (genotype *Mct8^+/−^*) to obtain mice deficient in both D2 and Mct8 (*Mct8D2KO*; genotype *Mct8^−/y^D2^−/−^*). To produce the male mice used in the experiments female *Mct8^+/−^D2^−/−^* were mated with male *Mct8^−/y^D2^−/−^* mice, producing *Mct8^+/y^D2^−/−^* (*D2KO*) and *Mct8^−/y^D2^−/−^* (*Mct8D2KO*) male littermates. The *D2KO* genotype was confirmed by PCR of tail DNA (38 cycles at 62°C annealing temperature) using the following primers: Reverse common: 5′- GTTTAGTCATGGAAGCAGCACTATG-3 forward *WT*-specific: 5′- CATGGCGTTAGCCAAAACTCATC-3′; forward *KO*-specific: 5′- CGTGGGATCATTGTTTTTCTCTTG-3′. The procedure generates a 400 bp fragment from the WT allele and a 450 bp fragment from the null allele.

### Induction of hypothyroidism and hyperthyroidism

Hypothyroidism was induced in adult male *WT* and *Mct8KO* mice by administering 0.02% 1-methyl-2-mercapto-imidazol (MMI, Sigma Chemical Co, St Louis, MO) plus 1% KClO_4_ *ad libitum* in the drinking water for 50 days before sacrifice. Hyperthyroidism was induced in *WT* mice by administering 10 µg T_3_/100 g BW daily in the drinking water, for the 9 days prior to sacrifice. Since this high dose of T3 was expected to suppress TSH and thyroid secretions, the mice received also in the drinking water a physiological dose of T_4_, 2 µg/100 g BW to maintain T_4_ concentrations within normal levels. The rational for this schedule was to keep normal T_4_ concentrations in the face of T_3_-induced hyperthyroidism, thus maintaining regulatory events due to extra genomic actions of T_4_ [34] or related to T_4_ to T_3_ conversion by D2 activity [35] This protocol has been validated earlier in experiments involving *WT* and *D3KO* mice. The mice treated in this way have highly increased T3 concentrations but normal T_4_, and unchanged brain D2 activity [36]. In all cases mice were killed between 9 a.m. to 12 a.m. after an intraperitoneal injection of a mixture of ketamine 100 mg/kg and medetomidine 0.1 mg/kg).

### Measurement of gene expression by quantitative polymerase chain reaction (qPCR)

The cerebral cortex was rapidly dissected out, frozen on dry ice, and kept at −80°C until RNA isolation using the Trizol procedure (Invitrogen, Carlsbad, CA). Complementary DNA was prepared from 250 ng of RNA using the High Capacity cDNA Reverse Transcription kit (Applied Biosystems, Foster City, CA). PCR was performed using a cDNA aliquot synthesized from 5 ng of RNA, with Taqman primers for *Hr* (hairless) and *D1* (type 1 deiodinase) and the Taqman Universal PCR Master Mix, No Amp Erase UNG (Applied Biosystems) on a 7900HT Fast Real-Time PCR System (Applied Biosystems). The PCR program consisted in a hot start of 95°C for 10 min, followed by 40 cycles of 15 sec at 95°C and 1 min at 60°C. PCRs were performed in triplicates, using the 18S gene as internal standard and the 2−^ΔΔCt^ method for analysis.

### Infusion protocol and extracts preparation for NMR spectroscopy

Mice were deeply anesthetized with 1–2% isoflurane in 1 L min^−1^ O_2_ through a nose cap, and infused through the jugular with (1-^13^C) glucose (8 µmol min^−1^ g^−1^) during 30 min. The physiological condition of the animal was followed throughout the experiment. Respiratory rate was monitored by a Biotrig system (Bruker Medical GmbH, Ettlingen, Germany), and body temperature was recorded using a rectal probe (Panlab, Barcelona, Spain). Body temperature was maintained at approximately 37°C using a thermostatic blanket and a temperature-regulated circulating water bath. At the end of infusion, brain metabolism was immediately stopped *in situ* by using a 5-kW microwave fixation system (Muromachi Kikai Co. Ltd., Tokyo, Japan). The brains were then rapidly removed from the skull and immediately frozen in liquid nitrogen. Perchloric acid extracts were prepared from the individual brains, neutralized with KOH, lyophilized, and resuspended in 99.9% ^2^H_2_O, as described previously [37], [38], prior to performing high-resolution ^13^C NMR spectroscopy.

### ^13^C Nuclear Magnetic Resonance Spectroscopy

High-resolution proton-decoupled ^13^C NMR spectra of brain extracts were obtained at 11.9 T (125.13 MHz, 25°C, pH 7.2) with a Bruker AVANCE 500WB NMR spectrometer using a commercial (5 mm) triple resonance probe (^1^H, ^13^C, ^2^H) optimized for direct ^13^C NMR detection. The acquisition conditions were: π/3 pulses; 30.0 kHz spectral width; 1.09 s acquisition time; 64k words data table; and 6.0 s recycling time. Proton decoupling was only gated during the acquisition using a broad band composite pulse decoupling sequence, and chemical shifts were calibrated with an external reference of dioxane (10% v/v, 67.4 ppm). Resonance assignments were based on literature values and on the addition of internal standards [37]. Spectra deconvolution and multiplet structures were analyzed using the PC-based (Intel Centrino Platform) NMR program, NUTS™ (Acorn, Freemont, CA).

The absolute amount of ^13^C incorporated in the different carbons was determined by comparison of the area of the corresponding ^13^C resonances with the unchanged *myo*-inositol resonance areas of each one of the perchloric acid extracts [39], [40]. This was possible since *myo*-inositol had a low turnover and did not become enriched after a 30 min (1-^13^C) glucose infusion independently of the thyroid status [38], providing a robust internal reference from which all ^13^C enrichments can be derived. The absolute amount of ^13^C incorporation in the different carbons and the fractional enrichment was determined as previously described [41], [42].

### Other procedures

The amino acid content of brain extracts was determined with an automatic amino acid analyzer Biochrom 20 (Pharmacia, Uppsala, Sweden) using a cationic exchange column and precolumn derivatization with ninhydrin [43]. Thyroid hormone determinations were performed as described [44]. Statistical calculations were performed using the Graph-Pad Prism software (http://www.graphpad.com/prism/).

## Results

### Disruption of the Mct8 gene leads to metabolic changes in the brain suggesting hyperthyroidism

To correlate the lack of Mct8 with altered thyroid hormone transport to the brain we compared the metabolic changes caused by the lack of a functional Mct8 protein with situations of thyroid hormone deficiency or excess. Our approach was to measure whole brain metabolism by ^13^C NMR spectroscopy using (1-^13^C) glucose as substrate. Thyroid hormones are involved in the developmental timing of expression of many enzymes of intermediary metabolism and in neurotransmitter systems [2] during the postnatal period. Neonatal hypothyroidism causes a delay in many cellular and molecular events, for example expression of myelin genes, which are then normalized in matured animals. Therefore in this study we used adult animals to detect permanent changes induced by Mct8 deficiency.

**Figure 1:**
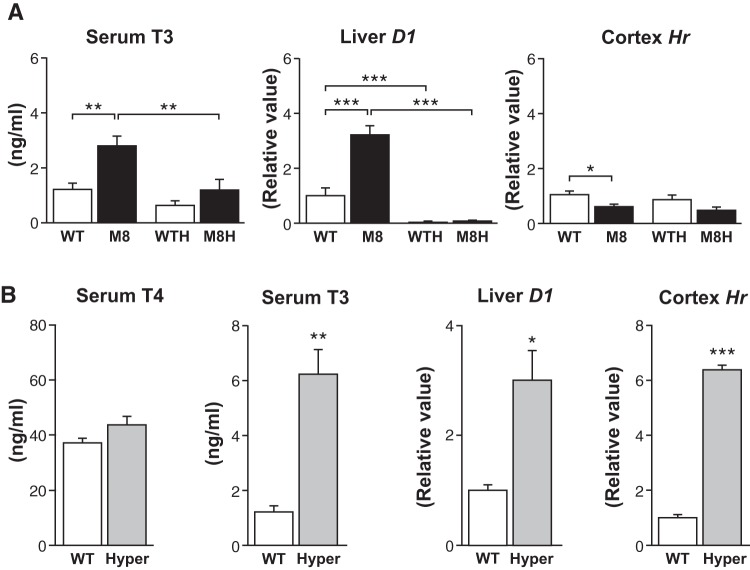
Serum thyroid hormone concentrations and expression of T_3_-dependent genes in liver and cerebral cortex. Panel A: Comparisons between untreated *Mct8KO* (M8, n = 5) and *WT* (n = 5) mice, and hypothyroid mice of either genotype (WTH and M8H, n = 5). Panel B: Comparisons between untreated *WT* mice (n = 4) and hyperthyroid mice (Hyper, n = 4) obtained by treatment with high doses of T_3_ and a physiological dose of T_4_. The data are mean ± SEM. Statistical significance between the groups was calculated by two-way ANOVA and the Bonferroni post-hoc test in the groups in panel A, and by the Student's *t*-test in panel B. * P<0.05; ** P<0.01; *** P<0.001.

First we compared Mct8 mutant mice with *WT* littermates and with hypothyroid mice of the same genotypes. Hypothyroidism was induced by treatment with the antithyroid compounds MMI and KClO_4_ which efficiently block thyroid hormone biosynthesis and produce general hypothyroidism. To assess the thyroidal status of the different groups of mice we measured serum T_3_ and the expression of 2 sensitive thyroid hormone target genes, *D1* in the liver and *Hr* in the cerebral cortex (Fig. 1A). Two-way ANOVA indicated a significant effect of genotype on serum T_3_ (*P* = 0.0045), which increased by around 100% in the *Mct8KO* mice. Antithyroid treatment lead also to significant changes of T_3_ (*P* = 0.00049), which decreased by around 50% in mice of both genotypes. Although serum T_4_ was not measured in these animals, similar groups showed the characteristic 30–50% reduction of T_4_ in the *Mct8KO* mice [26], [27], [45]–[47], and 85% reduction in the hypothyroid mice (not shown). There was a significant effect of genotype (*P* = 0.0023) and antithyroid drug treatment (*P*<0.0001) on *D1* expression, with an increase in the *Mct8KO* mice and a pronounced decrease in the hypothyroid mice of the two genotypes. As for *Hr* expression in the cerebral cortex, there was a significant effect of genotype, with a decrease in the *Mct8KO* mice (*P* = 0.0028), but no effect of treatment (*P* = 0.196).

**Figure 2:**
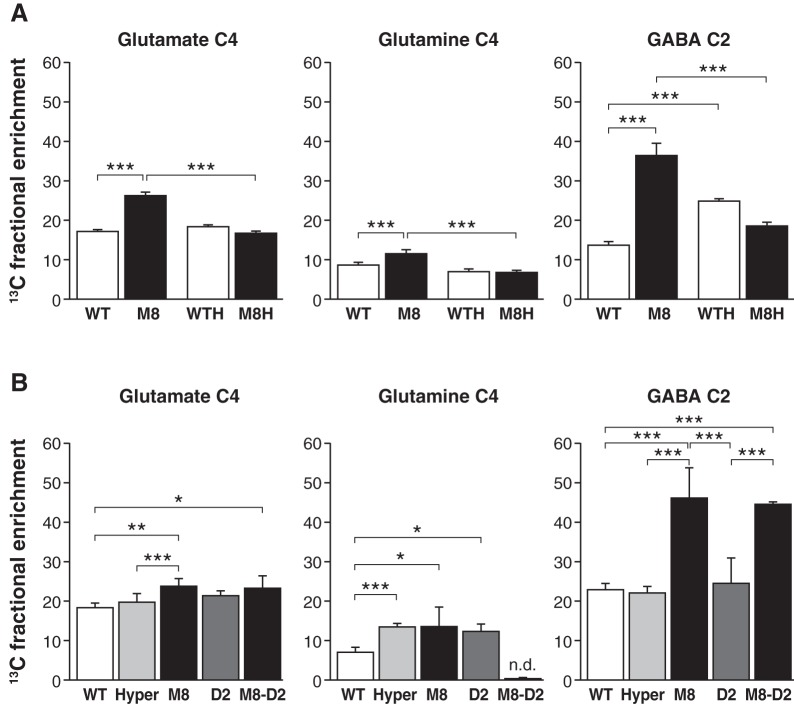
^13^C fractional enrichment into glutamate C4, glutamine C4 and GABA C2. Panel A: Comparisons between untreated *Mct8KO* (M8, n = 6) and *WT* (n = 6) mice, and hypothyroid mice of either genotype (WTH and M8H, n = 5). The data are mean ± SD. Panel B: Comparisons between untreated (WT, n = 6) and hyperthyroid (Hyper, n = 6) *WT* mice, *Mct8KO* mice (M8, n = 4), *D2KO* mice (D2, n = 4) and *Mct8D2KO* mice (M8-D2, n = 4). The data are mean ± SD. Statistical significance between the groups was calculated by two-way ANOVA and the Bonferroni post-hoc test in the groups in panel A, and by one-way ANOVA and the Tukey post-hoc test in the groups in panel B. * P<0.05; ** P<0.01; *** P<0.001.

Fig. 2A shows the effects of *Mct8* gene disruption and antithyroid treatment on the ^13^C fractional incorporation into glutamate C4, glutamine C4, and GABA C2 after infusion of (1-^13^C) glucose. There was a significant effect of genotype on the fractional enrichment of ^13^C into glutamate C4 (*P*<0.0001), glutamine C4 (*P* = 0.00275), and GABA C2 (P<0.0001), with increases in ^13^C labeling of the three metabolites in the mutant mice. Antithyroid treatment also led to differences in glutamate C4 (*P*<0.0001), glutamine C4 (*P*<0.0001), and GABA C2 (*P* = 0.0357) enrichments, which were mainly due to effects of hypothyroidism on the *Mct8KO* mice. The interaction between genotype and treatment was significant, indicating that mice from each genotype responded differently to antithyroid treatment. Treatment of *WT* mice with antithyroid drugs increased ^13^C incorporation into the GABA pool without affecting the glutamate or glutamine pools (Table 1, experiment 1). The same treatment was very effective on the *Mct8KO* mice, with a reduction in the incorporation of ^13^C into all metabolites, which were now similar values of *WT* mice. There was no difference in amino acids concentrations in the brain extracts (Table 1, experiment 1). In an attempt to define at the molecular level the mechanisms for these changes we measured the expression of relevant enzymes by qPCR: no effects of *Mct8* gene inactivation or of hypothyroidism were found on the expression of *Gad1* (glutamate decarboxylase), *Glud1* (glutamate dehydrogenase), *Atp1a3* (Na^+^K^+^-ATPase), *Gls* (glutaminase), *Glul* (glutamine synthetase), and *Gabat* (GABA transaminase), nor we found differences in the relative content of both forms of glutamate decarboxylase (GAD65 and GAD67) by western blotting (data not shown).

**Table 1:** Amino acids concentrations in brain extracts (μmol g^−1^ wet weight).

	Glutamate	Glutamine	GABA
Experiment 1			
*WT* (6)	6.35±0.80	3.15±0.72	1.32±0.15
*Mct8KO* (6)	6.60±0.35	3.52±0.72	1.51±0.27
*WT* Hypo (5)	5.97±0.89	2.90±0.25	1.25±0.16
*Mct8KO* Hypo (5)	6.55±0.95	3.30±0.12	1.40±0.34
Experiment 2			
*WT* (6)	5.83±1.12	2.66±0.54	1.26±0.23
*Mct8KO* (4)	6.56±1.23	3.60±0.78	1.40±0.34
*WT* Hyper (6)	6.25±0.45	3.36±0.65	1.38±0.11
*D2KO* (4)	6.66±0.49	3.23±0.90	1.29±0.22
*Mct8D2KO* (4)	5.94±0.67	3.43±1.01	1.34±0.19

The number of animals is given in the first column after the genotypes.

Data are mean ± SEM.

Assuming that the metabolic changes in the Mct8 mutant mice were due to thyroid hormone deficiency in the brain, in agreement with the deficient T_3_ transport and the decreased *Hr* expression, it was difficult to explain why they were normalized by blocking thyroid hormone synthesis. Paradoxically, the results rather seemed to indicate that the metabolic alterations in the brain of the *Mct8KO* mice were due to excess thyroid hormone.

### Effects of hyperthyroidism and of D2 inactivation

Prompted by the above results we next evaluated the effect of hyperthyroidism. Hyperthyroid *WT* mice were produced by treatment with high doses of T_3_ and a physiological dose of T_4_ for several days. This treatment led to a 6-fold increase of circulating T_3_ and normal concentrations of T_4_ (Fig. 1B). In agreement with the increased circulating T_3_, liver *D1* and cerebral cortex *Hr* expression were increased in the hyperthyroid mice 3- and 6-fold respectively. As shown in Fig. 2B, again the *Mct8KO* mice showed increased ^13^C fractional incorporation into glutamate C4, glutamine C4 and GABA C2. Hyperthyroidism increased ^13^C incorporation into glutamine, but had no effect on glutamate or GABA. Therefore, the changes induced by the absence of a functional Mct8 were not replicated in the hyperthyroid mice. There was no effect of hyperthyroidism on amino acids concentrations (Table 1, experiment 2).

In parallel to the effect of hyperthyroidism we also evaluated the effect of inactivating the *D2* gene. D2 is an astrocytic enzyme that generates T_3_ from T_4_. The activity of this enzyme is increased in the brain of the *Mct8KO* mice, and partially compensates for the lack of T_3_ uptake from the circulation [26], [27]. We have previously shown that *D2* gene inactivation in the *Mct8KO* mice induces a state of brain hypothyroidism on P21 similar to thyroid hormone deprivation [28]. To analyze the possible contribution of increased T_3_ production from T_4_ in the brain from *Mct8*KO mice we included in the study the mice deficient in D2 (*D2^−/−^*), and the double knockout mice for Mct8 and D2 (*Mct8^−/y^D2^−/−^*). Absence of D2 increased incorporation of ^13^C into glutamine but had no effect on glutamate and GABA. In mice deficient in Mct8 and D2 there was increased ^13^C incorporation into glutamate C4 and GABA C2, as in the *Mct8KO*. It was noteworthy that ^13^C labeling in glutamine C4 was not detected in these mice. Again, there was no difference in amino acids concentrations in the brain extracts (Table 1, experiment 2).

## Discussion

Previous studies on the effects of hypothyroidism on brain metabolism using NMR spectroscopy have been done in adult and neonatal rats. Adult onset hypothyroidism decreased metabolism of (1,2-^13^C_2_) acetate, and reduced the incorporation of the label into cerebral metabolites [38], indicating that hypothyroidism reduced the consumption of acetate by the glial cycle, and reduced its metabolism in the glial and neuronal TCA cycles. More recently, Martinez *et al*. [48] have also found that neonatal hypothyroidism decreases the flux of glucose into the glutamate, glutamine and GABA pools. In agreement with a depression of cerebral metabolism by hypothyroidism, phosphofructokinase and pyruvate kinase activities decrease in the cerebral hemispheres and cerebellum of hypothyroid rats [49].

In contrast, we found that disruption of the *Mct8* gene induces a generalized increased of neuronal metabolism. ^13^C incorporation from glucose to the glutamate, glutamine, and GABA pools was increased, without changes in absolute pool values. Glutamatergic activity appears to be coupled to glucose oxidation. Therefore, the data revealed an increased neuronal glucose oxidation through the TCA cycle. Thyroid hormone deprivation of the mutant mice normalized the altered parameters, suggesting that the alterations of brain metabolism were a consequence of excess of thyroid hormones. Indeed, some behavioral traits of *Mct8KO* mice have been recently interpreted as signs of brain hyperthyroidism [25]. However, this interpretation is difficult to reconcile with what is known on the thyroid status of the brain of *Mct8KO* mice. The brain of these animals is in a state of hypothyroidism partially compensated by increased local production of T_3_ due to the elevated activity of D2 [26]–[28]. This is the reason why on P21 most thyroid hormone dependent genes have normal expression in the cerebral cortex, with a few exceptions, including *Hr*, a sensitive marker of thyroid hormone action. In the present study we found that the expression of *Hr* was also decreased in the *Mct8KO* mice, in agreement with previous findings [28], [45].

It is known that tissues other than the brain are in a hyperthyroid state in *Mct8KO* mice. It might be possible that extracerebral, mainly hepatic (1-^13^C) glucose metabolism have contributed to the changes observed in the brain. However, extracerebral glucose metabolism (mainly hepatic) would generate mainly (3-^13^C) lactate, (3-^13^C) alanine or ^13^C labeled amino acids. The permeability of these secondary metabolites through the BBB is known to be smaller than that of (1-^13^C) glucose. At the high (1-^13^C) glucose concentrations used in our study it is safe to maintain that the ^13^C NMR results are dominated by the cerebral metabolism of (1-^13^C) glucose and that extracerebral sources of (3-^13^C) lactate and ^13^C amino acids do not contribute appreciably to the ^13^C patterns observed. It is also unlikely that the changes observed are secondary to changes in expression of glucose transporters, since at the high (1-^13^C) glucose concentrations used, glucose metabolism in the brain is known not to be limited by the transport capacity.

On the other hand, the metabolic changes induced by Mct8 deficiency are different from those observed after inducing hyperthyroidism by pharmacological means. Hyperthyroidism increased the ^13^C fractional incorporation into glutamine C4, but not into glutamate C4 and GABA C2 as in the *Mct8KO*. On the other hand, disruption of the *D2* gene did not alter the metabolic flux through glutamatergic and GABAergic neurotransmissions. *D2* encodes an enzyme responsible for the local production of T_3_ from T_4_ in brain. Under normal conditions, this pathway provides around 50% of brain T_3_. In mice with D2 deficiency, T_3_ from the circulation is enough to compensate the expression of many thyroid hormone-dependent genes. However, since the uptake of circulating T_3_ depends on the normal expression of Mct8, absence of both, D2 and Mct8 as in the *Mct8D2KO* mice, results in brain hypothyroidism [28]. In this situation, we found that the changes of glutamate and GABA ^13^C labeling were the same as in the single Mct8 deficiency. These results reduce the possibility that the lack of Mct8 would have produced an increased T_3_ production through D2 activity responsible for the metabolic changes.

At present we lack a mechanistic explanation for many of the effects of Mct8 inactivation or of other genetic manipulations such as the increased glutamine C4 in the *D2KO*, or its lack of detection in the *Mct8D2KO* mice. The molecular and cellular bases of the effects of *Mct8* inactivation remain to be clarified. Despite this, it is clear that the metabolic changes induced by Mct8 deficiency in the brain could not be correlated with the overall deficiency or excess of thyroid hormone supply to brain. We do not favor the explanation that Mct8 might perform thyroid hormone-independent actions in the brain [24]. For example, the possibility that Mct8 could transport a hitherto unidentified metabolite, important for brain metabolism, seems unlikely in view of the narrow substrate specificity of Mct8 [50].

The adult *Mct8KO* mice studied in this work might actually display a more pronounced brain hypothyroidism than that attained by adult onset antithyroid treatment of the *WT* mice. Indeed, *Hr*, a very sensitive T_3_ target gene was altered in the brain of adult *Mct8KO* mice and not in the adult hypothyroid *WT* mice. This is in agreement with the lower sensitivity of the adult brain in comparison to the postnatal brain, to hypothyroidism-related changes of gene expression [51]. (This study described the results of microarray analysis of the adult rat striatum comparing hypothyroid rats with hypothyroid rats treated with T_3_, either in a high single dose (SD) to induce hyperthyroidism, or with daily replacement doses (RD) to reach euthyroidism. Whereas the SD treatment resulted in expression changes of many genes, the result of RD treatment was modest. In particular *Hr* expression was increased in the SD groups and did not change in the RD groups. These experiments were compatible with the view that the adult rat striatum is more responsive to hyperthyroidism than to hypothyroidism in terms of gene expression). The Mct8 deficiency in the *Mct8KO* mice was present from early embryonic stages in contrast to the adult-onset hypo or hyperthyroidism in the *WT* used for comparison. Brain responses to thyroid hormones may have been altered permanently in the *Mct8KO* mice, which show signs of T_3_ deficiency and excess at different stages of life [25]–[27], [46]. Therefore, it is possible that the lack of Mct8 might have triggered a compensatory increase of brain metabolism as a response to the sustained hyperthyroidism in the liver and other tissues or to the chronic impairment of T_3_ uptake in the brain. Further investigation into the molecular mechanisms for this metabolic response will be important to define the physiopathology of the brain alterations present in MCT8 deficiency.

**Figure 3:**
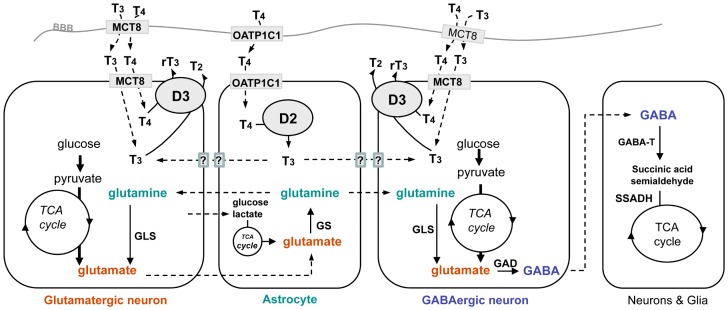
Thyroid hormone transport and metabolism, and neurotransmitter cycling in astrocytes and neurons. T_4_ crosses the blood-brain barrier and is delivered to the astrocytes through the Oatp1c1 transporter, a 12-transmembrane protein localized in the micro capillary endothelial cells, and in the astrocytic end-feet. Astrocytes express the type 2 deiodinase (D2), localized in the endoplasmic reticulum. This enzyme catalyzes the phenolic ring deiodination of T_4_ to produce the active hormone T_3_. T_3_ acts genomically on astrocytes and neurons. The transporters facilitating the passage of T_3_ from astrocytes to neurons have not been defined. Most likely they consist of a combinatorial mixture of different transporters depending upon age and cell type. T_3_ and T_4_ cross the blood-brain barrier through Mct8, reaching the neurons from the interstitial fluid through Mct8, and possibly by other transporters. Neurons express the type 3 deiodinase (D3) a plasma membrane protein, which catalyzes the tyrosil ring deiodination of T_4_ and T_3_ to produce the inactive metabolites rT_3_ and T_2_, respectively. In astrocytes and neurons, glutamate is produced in the TCA cycle after glycolysis. Astrocytes can also use the lactate produced by neurons as substrate. Astrocyes express the enzyme glutamine synthetase (GS), which produces glutamine from glutamate. Neurons express the phosphate-activated glutaminase (GLS), which converts glutamine back to glutamate. In GABAergic neurons the enzyme glutamic acid decarboxylase (GAD) converts glutamate to GABA. GABA is degraded in astrocytes and neurons by GABA transaminase (GABA-T), with the formation of succinic acid semialdehyde. This metabolite enters the TCA cycle after conversion to succinic acid by succinic semialdehyde dehydrogenase (SSADH). Membrane transporters for glucose and neurotransmitters have not been added for clarity.

Finally, figure 3 illustrates the parallelisms between thyroid hormone and neurotransmitter transport and metabolism, indicating specifically the cooperation between astrocytes and neurons in both processes. In late postnatal and adult WT mice, about 50% of the T_3_ present in the brain parenchyma originates in the astrocytes by the D2-catalyzed phenolic ring deiodination [33]. The rest of brain T_3_ mostly enters the brain from the circulation through Mct8 present in the BBB. Thyroid hormones may also reach the brain parenchyma in limited amounts through the cerebrospinal fluid [52]. On the other hand, circulating T_4_ crosses the BBB through the specific T_4_ transporter Oatp1c1, in addition to Mct8. Oatp1c1 is also present in the astrocytic end-feet, facilitating direct delivery of T_4_ to the D2-expressing astrocytes. In Mct8KO animals, the absence of T_3_ transport through the BBB is compensated by an increased production of T_3_ from T_4_ in the astrocytes. The absence of Mct8 apparently does not result in the restriction of astrocytic T_3_ delivery to neurons possibly through alternative transporters [28]. However, as shown in this paper, the absence of Mct8 leads to dramatic alterations of neurotransmitter metabolism, with increased cycling of glutamate, glutamine, and GABA. At present, it is uncertain whether this is a consequence of the changes in the T_4_ and T_3_ cellular fluxes brought about by the absence of Mct8-mediated transport, and/or whether they are related to changes in the expression of enzymes. Indeed, changes in the intercellular flux of T3 caused by D2 deficiency lead to selective changes in genes regulated negatively by thyroid hormone by as yet unknown mechanisms [28], [36]. On the other hand we failed to detect changes in *Gls*, *Atp1a3*, *Glud1*, *Gad1*, *Glul*, and *Gabat* expression using the whole cerebral cortex, but changes affecting in a restrictive fashion specific cell groups or regions cannot be discarded. In relation to the increased rate of GABA production, it is worth mentioning that succinic semialdehyde dehydrogenase (SSADH) deficiency in humans and mice, with accumulation of GABA and gamma-hydroxybutyrate (GHB) causes severe neurodevelopmental impairment [53]. Whether an increased GABA cycling contributes to the phenotypic manifestations of MCT8 deficiency in humans is an intriguing possibility deserving further exploration.

In summary, Mct8 deficiency in mice results in profound alterations of brain metabolism, consisting of increased oxidative metabolism and neurotransmitter cycling which could not be correlated with thyroid hormone deficiency or excess. While the mechanisms leading to this situation are still unclear, these data could be relevant to explain the physiopathology of the profound neurological impairment present in MCT8 mutations.
